# Superconductivity at Interfaces in Cuprate‐Manganite Superlattices

**DOI:** 10.1002/advs.202301495

**Published:** 2023-05-10

**Authors:** Nicolas Bonmassar, Georg Christiani, Tobias Heil, Gennady Logvenov, Y. Eren Suyolcu, Peter A. van Aken

**Affiliations:** ^1^ Max Planck Institute for Solid State Research Heisenbergstraße 1 70569 Stuttgart Germany; ^2^ Department of Materials Science and Engineering Cornell University Ithaca NY 14853 USA

**Keywords:** chemical potential, electron energy loss spectroscopy, Jahn–Teller effect, molecular beam epitaxy, scanning transmission electron microscopy, superconductivity, ultrathin films

## Abstract

One of the unsolved problems for using high‐*T*_c_ superconducting cuprates for spintronic applications are the short coherence lengths of Cooper pairs in oxides (a few Å), which requires atomically sharp and defect‐free interfaces. This research demonstrates the presence of high‐*T*_c_ superconducting La_1.84_Sr_0.16_CuO_4_ in direct proximity to SrLaMnO_4_ and provides evidence for the sharpness of interfaces between the cuprate and the manganite layers at the atomic scale. These findings shed light on the impact of the chemical potential at the interface of distinct materials on highly sensitive physical properties, such as superconductivity. Additionally, this results show the high stability of ultrathin layers from the same K_2_NiF_4_‐type family, specifically one unit cell of Sr_2−_ *_x_* La*_x_* MnO_4_ and three unit cells of La_1.84_Sr_0.16_CuO_4_. This work advances both the fundamental understanding of the proximity region between superconducting cuprates and manganite phases and the potential use of oxide‐based materials in quantum computing.

## Graphical abstract

Tuning the sharpness of the interface results from tuning the chemical potential at interfaces. Differences in chemical potential lead to local structural distortions, cation intermixing, and the formation of parasitic phases. Therefore, even small distortions or non‐stoichiometry can alter the physical properties at the nano‐scale.

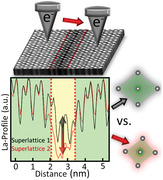

## 1 Introduction

The study of cuprate‐manganite interfaces has gained significant attention in recent years for its potential in oxide‐based quantum computing,^[^ ^1^, ^2^, ^3^ ^]^ and its impact on proximity‐,^[^ ^4^ ^]^ and exchange bias effects.^[^ ^5^ ^]^ The goal of creating a superconducting *π*‐qubit that is fully isolated from its surroundings is a topic of ongoing research.^[^ ^6^, ^7^, ^8^, ^9^, ^10^, ^11^ ^]^ However, a major obstacle in achieving this goal is the short coherence lengths (*ξ*) of Cooper pairs in oxide materials, which are only a few tens of Å and a few Å for *ξ*_in‐plane_ and *ξ*_out‐of‐plane_, respectively.^[^ ^12^ ^]^ Due to these extremely short coherence lengths, the control of superconductivity at the interface in direct proximity to the tunnel barrier is of utmost importance.^[^ ^13^, ^14^ ^]^

It is well known that superconductors are highly susceptible to changes in their apical oxygen distances or small off‐stoichiometries in their chemical distribution.^[^ ^15^, ^16^ ^]^ Even a thin layer of one half‐unit cell of non‐superconducting material at the top and the bottom interface can inhibit Josephson coupling between the two superconductors without considering the actual tunnel barrier. To minimize structural or chemical defects at the interface, choosing a tunnel barrier should not only be based on structural compatibility, but also on the chemical potential difference between the tunnel barrier and the superconducting material.^[^ ^17^ ^]^ It is also known that intermixing occurs at the interface between two materials with different elements.^[^ ^18^, ^19^ ^]^ A minimization of the difference of the chemical potential at the interface leads to less intermixing. Therefore, controlling the chemical potential difference at the interface also means controlling the physical properties.

In this work, we study the effect of minimizing the chemical potential between optimally doped superconducting La_1.84_Sr_0.16_CuO_4_ (LSCO) and insulating Sr_2−_ *_x_* La*_x_* MnO_4_ phases (with either *x* = 0, SMO, or *x* = 1, LSMO) by doping Sr_2_MnO_4_ with La. To achieve high quality interfaces, we use ozone assisted molecular beam epitaxy (MBE) monitored by in situ reflection high energy electron diffraction (RHEED).^[^ ^20^, ^21^, ^22^ ^]^ This method allows us to control the growth of our samples and ensure that the interfaces are of the highest quality. For a thorough structural and chemical characterization of the interfaces at the atomic scale, we utilize several scanning transmission electron microscopy (STEM) techniques, such as annular bright‐field (ABF) imaging, high‐angle annular dark‐field (HAADF) imaging, electron energy‐loss spectroscopy (EELS), and energy‐loss near edge structure (ELNES) analyses. These techniques enable us to visualize and analyze the interfaces at the atomic level and gain a deep understanding of their structure and composition. Distortions of superconducting CuO_6_‐ and insulating MnO_6_‐octahedra are a result of the Jahn–Teller effect and can be imaged using ABF.^[^ ^23^ ^]^ As structural distortions can have a profound influences on the electronic configuration of the material, we show the unoccupied density of states (DOS) to highlight the complex orbital occupation in the interfacial CuO_6_‐ and the MnO_6_‐octahedra.

Finally, we show how to control superconductivity in the proximity region to a tunnel barrier by manipulating the chemical potential at the interface. Our findings provide valuable insight into the relationship between the chemical potential and superconductivity and can help further the development of oxide‐based quantum computing.

## 2 Results and Discussion

**Figure 1:**
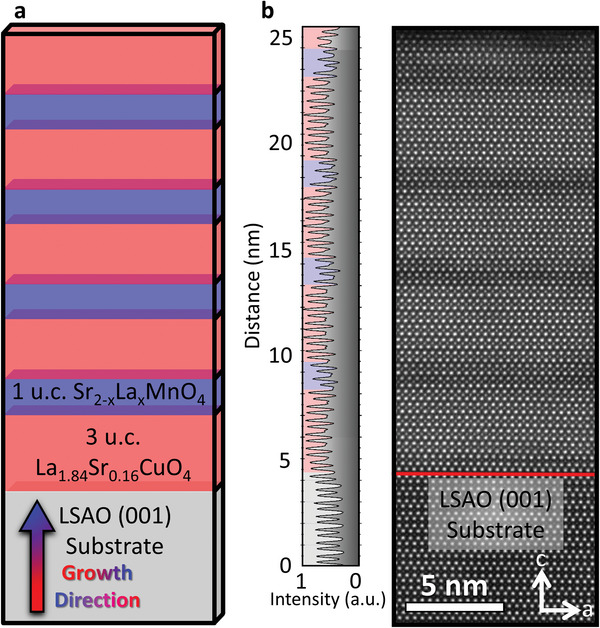
Overview of the as‐grown samples. a) Schematic of the designed heterostructures, with *x* = 0 and *x* = 1. b) Left: HAADF intensity profile integrated from the HAADF image. Red and blue background account for LSCO and LSMO layers, respectively. Right: HAADF overview image of the SL consisting of LSCO and LSMO. Red bar points out the interface from the substrate to the first LSCO layer.

In this study, we grow superlattices (SL) of ultrathin superconducting and insulating layers with a total thickness of ≈25 nm. We designed two different SL, each consisting of a four‐fold repetition of three unit cells LSCO and one unit cell SMO‐ or LSMO‐layers, respectively (**Figure** **1**a,b; Figure S1, Supporting Information). An overview of the high quality SL with alternating LSCO and LSMO layers is shown in the HAADF image in Figure 1b and similar results are presented in Figure S2 (Supporting Information) for the SL with SMO layers. The image shows an alternating pattern of thick bright LSCO layers and thinner dark LSMO layers with atomic resolution, starting from the substrate (red bar). The black profile on the left in Figure 1b highlights the HAADF intensity along the SL. The topmost unit cell of the LSCO layer at the surface was affected by environmental influences like moisture and is observed as a damaged layer on top of the heterostructure. To preserve the continuity of the interfaces, the last three unit cells of LSCO on top of the SL are deposited as a protective layer.

**Figure 2:**
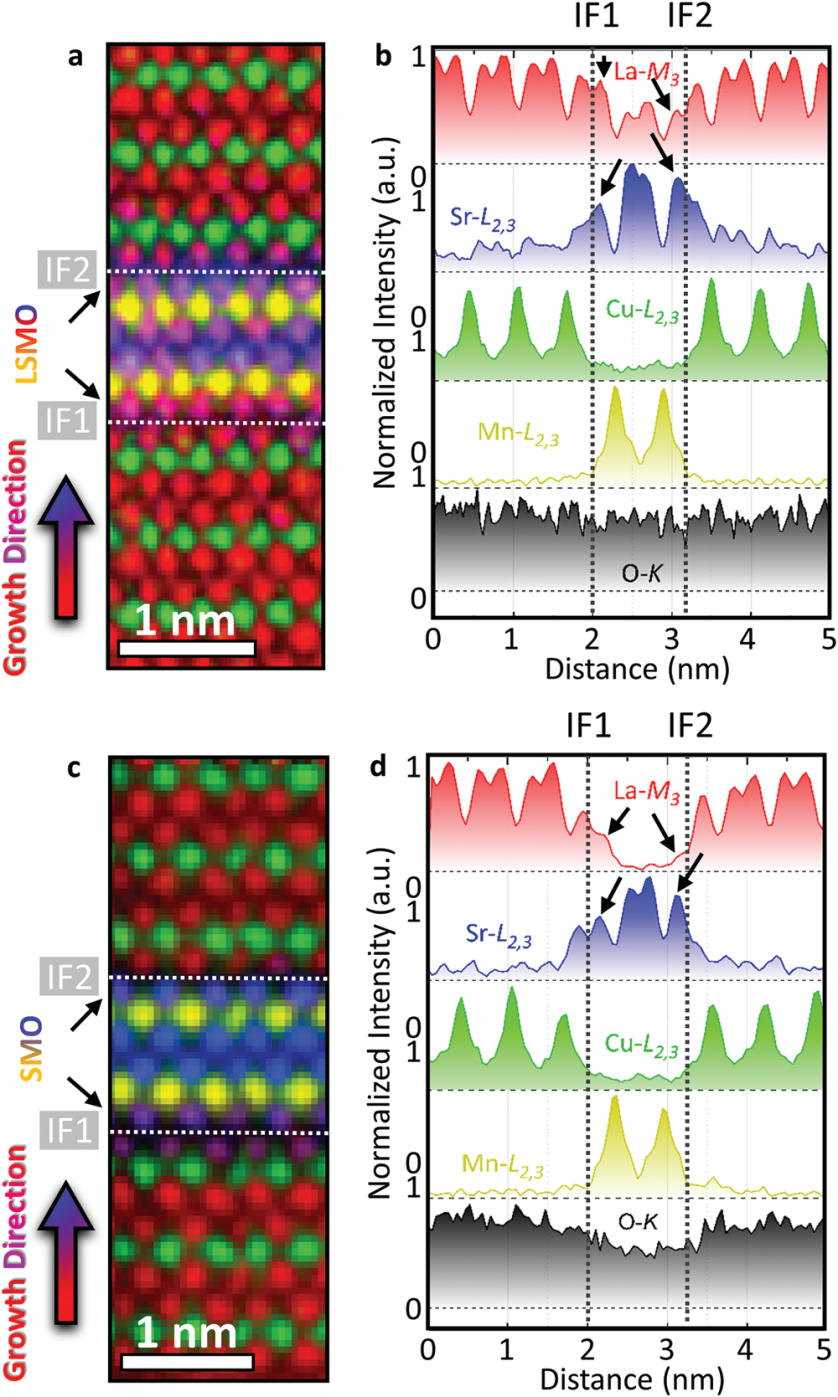
Analysis of chemical distribution of elements. a,c) EELS elemental mapping and b,d) EELS elemental profiles across two interfaces, IF1 and IF2, highlighted as dashed white lines for the both superlattices, respectively. The small black arrows highlight differences in La and Sr content within one unit cell of a) LSMO and b) SMO. The color code for the EELS mapping and the elemental profiles is red: La, blue: Sr, green: Cu, yellow: Mn, and black: O.

We investigate the two interfaces (IF1 and IF2 highlighted by white dashed lines in **Figure** **2**a–c) of the cuprate‐manganite systems using STEM combined with EELS for chemical mapping, which were denoised for a better guide to the eye for the reader (see Figure S4, Supporting Information for raw spectrum images). The extracted profiles are obtained from raw data and show one to two atomic layer La/Sr intermixing at both sides of the interfaces (see black arrows in Figure 2b,d). Notably, no intermixing of Cu and Mn atoms was observed within the experimental sensitivity of our instruments, despite the thickness of only one unit cell of LSMO and SMO. Both samples show clear signs of Sr segregation (see black arrows below IF2) and more La is detected in the bottom part of the respective unit cell (see black arrows on top of IF1). This segregation is due to the atomic weight differences between La and Sr and has been discussed elsewhere.^[^ ^24^ ^]^ Note that the SMO layer displays a stronger intermixing of Sr and La cations as compared to the LSMO layer. The effect of this unique type of intermixing on the oxidation state of the Mn atoms will be further explored later on using atomically‐resolved fine‐structure analyses of each MnO_6_‐octahedron. Finally, oxygen vacancies could be detected in the SMO layers, but not in the LSMO layers.

**Figure 3:**
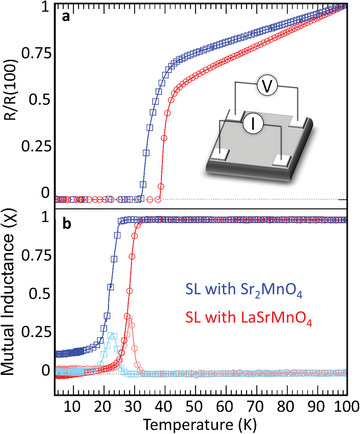
Physical properties of both samples. a) Resistance and b) mutual inductance versus temperature curves in blue squares and red circles for the SL with SMO and LSMO, respectively. The inset in (a) shows the setup for the used four‐point van der Pauw geometry. Imaginary parts of the mutual inductance measurements are depicted in lighter blue and lighter red colors, respectively.

Both SLs show high‐*T*_c_ superconductivity, despite using ultrathin layers of LSCO as indicated by resistance (**Figure** **3**a) and mutual inductance (Figure 3b) measurements. The resistance curves reach zero resistance at 31 and 38 K for the sample with SMO and LSMO, respectively. The SL with LSMO shows a sharper transition. Mutual inductance measurements reach a full diamagnetic response signal at 25 and 32 K in the SL with SMO and LSMO, respectively. The differences in critical temperatures between mutual inductance and resistance measurements are typically attributed to a percolation origin of superconductivity resulting from the proximity effect.^[^ ^25^, ^26^ ^]^ The ultrathin layers of LSCO used in this study are susceptible to exhibiting different critical temperatures due to small differences in the structural and/or electronic configuration,^[^ ^27^, ^28^ ^]^ such as CuO_6_ octahedral tilts, elongations, or compressions. Furthermore, the ultrathin superconducting LSCO layers are sandwiched between non‐superconducting manganites, which can lead to a buckling of the CuO_2_ planes.^[^ ^29^ ^]^ In addition, the in situ RHEED pattern indicates the presence of four streaks (Figure S3, Supporting Information),^[^ ^30^ ^]^ which depict a 5×5 super structured oxygen ordering,^[^ ^20^ ^]^ typically associated with the presence of superconductivity in optimally doped LSCO. Our findings allow us to combine the microscopic information of sharp LSCO structures with the macroscopic evidence for a clean and optimally doped LSCO unit cell in direct proximity to an insulating phase.

**Figure 4:**
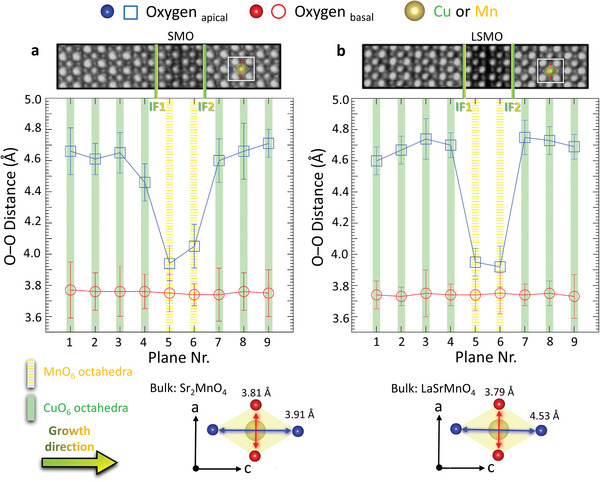
Apical and basal oxygen distances for the SL with a) SMO and with b) LSMO. The top parts are overlays of HAADF and iABF images for simultaneous visualization of oxygen and A, and B site cation positions. The green arrow points out the growth direction and the two octahedra depict the apical and basal oxygen distances for SMO and LSMO bulk material.^[^ ^32^, ^37^ ^]^ Error bars arise from two times the standard deviation of atomic‐column positions within the same plane.

In a first step, we demonstrate the presence of typical anti‐Jahn–Teller distorted LSCO layers in close proximity to the formally insulating manganite phase by measuring the oxygen–oxygen distances in both basal and apical directions.^[^ ^31^ ^]^ We utilize ABF imaging to determine the basal and apical oxygen distances of the different CuO_6_‐ or MnO_6_‐octahedra. **Figure** **4** shows overlays of inverted ABF (iABF) and HAADF images and their analyses. Additionally, we distinguish between different planes inside the CuO_6_‐ (green bars) and MnO_6_‐octahedra (yellow bars). The basal oxygen distances (red circles) remain constant for the manganites and the cuprates in both samples (SMO in Figure 4a, LSMO in Figure 4b) and are the same as in the LSAO (001)‐oriented substrate, i.e., 3.75 Å.^[^ ^32^ ^]^ Due to the total thickness of only 25 nm, this holds true for all layers, even for the ones that are far away from the substrate. The apical oxygen distances (blue squares), show a significant decrease when transitioning from LSCO layers (4.6 and 4.75 Å) to manganite layers (4.0 and 3.9 Å). Furthermore, we observe a sharper decrease at interface 1 (IF1) and a sharper increase at interface 2 (IF2) of the CuO_6_ apical oxygen distances in the SL with LSMO compared to the SL with SMO. Additionally, the apical oxygen distances of only 3.9 Å for the ultrathin LSMO layers are notably different from their bulk counterparts, which have a distance of 4.5 Å.^[^ ^31^ ^]^ This indicates the presence of strongly compressed MnO_6_‐octahedra. These longitudinal compressions alter the electronic configuration of the materials by raising the 3*dz* ^2^ − *r* ^2^ (out‐of‐plane) states, resulting in more occupied 3*dx* ^2^ − *y* ^2^ (in‐plane) orbitals.^[^ ^33^ ^]^ The same compression compared to the bulk material has been detected in HAADF results, c.f. Figure S5 (Supporting Information). In addition, a reduction of the cell volume has been detected in the SMO layer, which is in agreement with the detection of oxygen vacancies in this phase. Such strong effects can even result in completely different physical properties in the strained thin film compared to the unstrained bulk material.^[^ ^34^, ^35^, ^36^ ^]^

**Figure 5:**
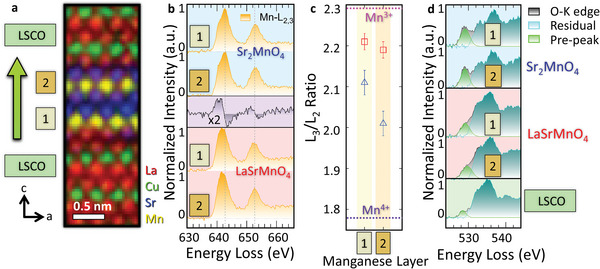
Electronic configuration of the one unit cell thick manganite layers in direct proximity to two superconducting LSCO layers. a) EELS elemental mapping serves as a guide to the eye for the different regions where the following EEL spectra have been integrated. The green arrow points out the growth direction. b) Mn‐*L*_2,3_ edges (orange) of the first and second monolayer of the respective unit cells for SMO (blue background) and for LSMO (red background). The spectrum with the purple background in (b) represents the difference spectrum between the sum of spectra 1 and 2 for LSMO and SMO, which highlights the earlier onset of the Mn‐*L*_2,3_ edge in LSMO compared to SMO. c) Mn‐*L*_2,3_ ratios of the respective manganese layers with Mn^3+^ and Mn^4+^ reference ratios obtained from LaMnO_3_ and SrMnO_3_, respectively. d) O‐*K* edges (black) of the first and second monolayer of the respective unit cells SMO (blue background) and LSMO (red background), and sum of the top and bottom O‐*K* spectra for LSCO monolayers (green background). Each pre‐peak is fitted with a Gaussian function (green) and the residual signal of the O‐*K* edge is depicted in turquoise.

The impact of these structurally distorted MnO_6_‐octahedra on the electronic configuration of the manganite layers is characterized by utilizing ELNES analyses with atomic resolution (**Figure** **5**). To gain a deeper understanding of the individual unoccupied DOS of the MnO_6_‐octahedra, we divide the crystallographic unit cell of the Sr_2−_ *_x_* La*_x_* MnO_4_ phases into first and second monolayers as shown in Figure 5a and perform ELNES analyses on raw data. Then, we compare the individual Mn‐*L*_2,3_ edges of SMO (blue background, Figure 5b) with the Mn‐*L*_2,3_ edges of LSMO (red background, Figure 5b). A difference spectrum (Figure 5b, purple background, LSMO spectrum minus SMO spectrum from position 1) of the two manganite phases highlights the earlier onset of the Mn‐*L*_3_ edge in LSMO compared to SMO. The Mn‐*L*_3_/*L*_2_ ratios are consistently lower for Mn atoms that are located in the second monolayer, indicating higher oxidation states of these Mn atoms. The Mn‐*L*_3_/*L*_2_ ratios are calculated by the method of Tan et al. in Figure 5c.^[^ ^38^ ^]^ The reference values for the Mn‐*L*_3_/*L*_2_ ratios of Mn^3+^ and Mn^4+^ have been determined from bulk SrMnO_3_ and LaMnO_3_. A more pronounced difference in valence states is observed in SMO, whereas only a small difference in valence states is observed in LSMO. Finally, the fitted Gaussian functions in the pre‐edge region of the O‐*K* edges (Figure 5d) highlight a smaller pre‐peak in La‐doped LSMO compared to SMO, indicating a lower Mn valence in the LSMO layer. This finding qualitatively confirms the results from the Mn‐*L*_2,3_ edge analyses, where a lower oxidation state of Mn atoms in LSMO is detected. However, an electronic differentiation of the two separate MnO_6_‐octahedra of the O‐*K* pre‐peak was not possible in contrast to the *L*_3_/*L*_2_ ratio determination, where small differences could be detected. Furthermore, the O‐*K* edge pre‐peak (mobile carrier peak) at 528.8 eV of the CuO_6_‐octahedra (green Gaussian fit with green background in Figure 5d) together with the high apical oxygen distance of 4.75 Å (Figure 4b) indicates superconducting bottom and top CuO_6_‐octahedra that are separated by only one unit cell thick LSMO. Note that, the O‐*K* edge pre‐peak in the LSCO layer, which is induced by Sr‐doping of a Mott insulator (La_2_CuO_4_) resulting in the introduction of mobile carriers, should not be confused with the O‐*K* edge pre‐peak of other antiferromagnetic insulators like bulk LSMO and bulk SMO that stay an insulator despite exhibiting a pre‐peak in the O‐*K* edge.^[^ ^39^ ^]^

## 3 Conclusion

In summary, this is the first report about SLs consisting of only one unit cell thick SMO and LSMO layers between three unit cell thick superconducting LSCO layers. We deliberately focused on completely disentangled CuO_6_ and MnO_6_ octahedra, to avoid any interfacial hybridization of Cu 3*d* and Mn 3*d* orbitals, resulting in chemically sharp and superconducting interfaces. Notably, both LSMO and SMO deviate from their formal valence states of pure Mn^3+^ and Mn^4+^, respectively. However, the deviation from the formal pure Mn^3+^ state for LSMO lies within the possible stoichiometry variations that can originate from the calibration of metal fluxes (≈5% off stoichiometric) prior to the MBE growth. The deviation from the formally pure Mn^4+^ in the SMO layer is also due to the formation of oxygen vacancies in this phase, thereby lowering the Mn valence to a mixed Mn^4+^/Mn^3+^ state. On top of that, both manganite layers show clear signs of intermixing with neighboring La cations that ultimately alter the oxidation state of the Mn atoms. A stronger effect of the asymmetric cationic distribution on the Mn valence could be detected in the SMO layer compared to the LSMO layer (c.f. Figures 2b,d and 5c). We attribute this finding specifically to the higher chemical potential between LSCO and SMO as compared to the chemical potential between LSCO and LSMO. Additionally, a 113‐type parasitic phase can be detected in the SL consisting of SMO as indicated in Figure S2a (Supporting Information). These differences between the intermixed SMO phase and the more stable LSMO phase indicate the importance of the chemical potential between two phases on both sides of the interface. The top and the bottom LSCO layers in direct proximity to LSMO are less affected regarding their apical oxygen distances and Sr content as compared to the LSCO layers next to SMO. Differences in Sr/La intermixing that directly influence the apical oxygen distances and defect formation (parasitic 113‐type phase in SL with SMO) account for the higher *T*_c_ in both resistance and mutual inductance measurements of the SL with La‐doped LSMO.

Structurally, most materials with similar crystallographic a and b axes will match perfectly at the interface when epitaxially grown. However, this does not guarantee chemical perfectness, the absence of defects or the apical oxygen distances required for certain physical properties, such as superconductivity. In fact, the first layers of superconducting materials on top of the substrate or layered structures are often referred to as “dead layers” due to the absence of superconductivity in these layers.^[^ ^28^, ^40^ ^]^ This is particularly important for oxide‐based superconducting materials, as the coherence lengths of Cooper pairs in oxide materials are smaller as in superconducting metals. Therefore, it is crucial to minimize the chemical potential at the interface for the realization of future high‐*T*_c_ superconducting oxide‐based spintronic.

## 4 Experimental Section

### Oxide‐MBE Growth

LSCO‐(L)SMO heterostructures consisting of three unit cells LSCO and one unit cell (L)SMO were grown by molecular beam epitaxy (oxide MBE) on a LSAO (001) substrates (CrysTec GmbH) using ozone‐assisted‐MBE (DCA Instruments). The deposition conditions during the growth were ≈1∙10^−5^ Torr (under an oxidizing atmosphere consisting of ozone, radical‐ and molecular oxygen) and 640°C (pyrometer temperature). Each growth was monitored by in situ RHEED to assure for atomic layer‐by‐layer growth.

### Transport Measurements and X‐Ray Diffraction (XRD)

Resistance (*R*) measurements with alternative direct currents of ±30 µA were carried out in four‐point‐probe configuration (Van der Pauw) to verify superconductivity of the superlattices. Mutual inductance (MI) measurements were employed in a two‐coil configuration (parallel geometry) with an alternative current of 50 µA and a frequency of 1000 Hz. All temperature (*T*) dependent measurements were controlled by a motorized custom‐designed dipstick (*T* change rate < 0.1 K s^−1^) and the temperature was varied from room temperature to 5K. Out‐of‐plane XRD measurements were performed to check the general macroscopic quality of the sample. The diffractometer was equipped with a Cu‐K_*α*_ source (Bruker D8 Cu‐K_*α*1_ = 1.5406 Å).

### Scanning Transmission Electron Microscopy

Electron‐transparent specimens were prepared by tripod‐wedge polishing. A subsequent precision ion polishing system (PIPS II, Model 695) equipped with a liquid nitrogen filled cooling stage, to ensure for a safe sample preparation without severely damaging the sample, using Ar^+^ ions thinned the sample down to <20 nm thickness. All STEM analyses were performed using a JEOL JEM‐ARM200F STEM equipped with a cold‐field emission gun, a probe *C*_s_‐corrector (DCOR, CEOS GmbH) and a Gatan GIF Quantum ERS electron energy‐loss spectrometer equipped with a Gatan K2 direct electron‐detection camera. EELS and STEM results were collected at a convergence semi‐angle of 22 mrad resulting in a probe size of 0.8 Å. For annular dark‐field (ADF) imaging, the collection‐angle range was 87–209 mrad. EELS data were acquired at a collection semi‐angle of 87 mrad. A pixel dwell time of 3.7 ms and an energy dispersion of 0.5 eV channel^−1^ (resulting in an energy resolution of <1 eV) were used for the EELS elemental mapping and profiles, whereas for the Mn‐*L*_3_/*L*_2_ ratio determination a dispersion of 0.25 eV channel^−1^ was used (resulting in an energy resolution of ≈0.5 eV). Principle component analyses (PCA) were applied to reduce the noise for the color‐coded RGB maps in 2a,c and 5a. After PCA utilizing ten components, multiple linear least square (MLLS) fittings were performed on the PCA treated SIs as described elsewhere.^[^ ^40^ ^]^ The elemental profiles and all ELNES spectra with their subsequent analyses regarding the Mn‐*L*_3_/*L*_2_ ratios were extracted from raw data.

## Conflict of Interest

The authors declare no conflict of interest.

## Supporting information

Supporting Information

## Data Availability

The data that support the findings of this study are available from the corresponding author upon reasonable request.
