# Supplementary material for: Superconductivity at Interfaces in Cuprate‐Manganite Superlattices
Source: Adv Sci (Weinh). 2023 May 10;10(21):2301495. doi: 10.1002/advs.202301495 (PMC10375126; doi:10.1002/advs.202301495)
Supplement: Supplementary file 1 — Supporting Information [file ADVS-10-2301495-s001.pdf]

ADVANCED SCIENCE
Open Access

# Supporting Information

for *Adv. Sci.*, DOI 10.1002/advs.202301495

## Superconductivity at Interfaces in Cuprate-Manganite Superlattices

*Nicolas Bonmassar*, Georg Christiani, Tobias Heil, Gennady Logvenov, Y. Eren Suyolcu* and Peter A. van Aken*

# Supporting Information

**Supporting Information: Control of Superconductivity at Interfaces in Cuprate-Manganite Superlattices**

*Nicolas Bonmassar*, Georg Christiani, Tobias Heil, Gennady Logvenov, Y. Eren Suyolcu*, and Peter A. van Aken*

N. Bonmassar, G. Christiani, T. Heil, G. Logvenov, Y. E. Suyolcu, P. A. van Aken

Max Planck Institute for Solid State Research, Heisenbergstraße 1, 70569 Stuttgart, Germany

Y. E. Suyolcu

Department of Materials Science and Engineering, Cornell University, Ithaca, NY 14853, USA.

E-mail: n.bonmassar@fkf.mpg.de and eren.suyolcu@fkf.mpg.de

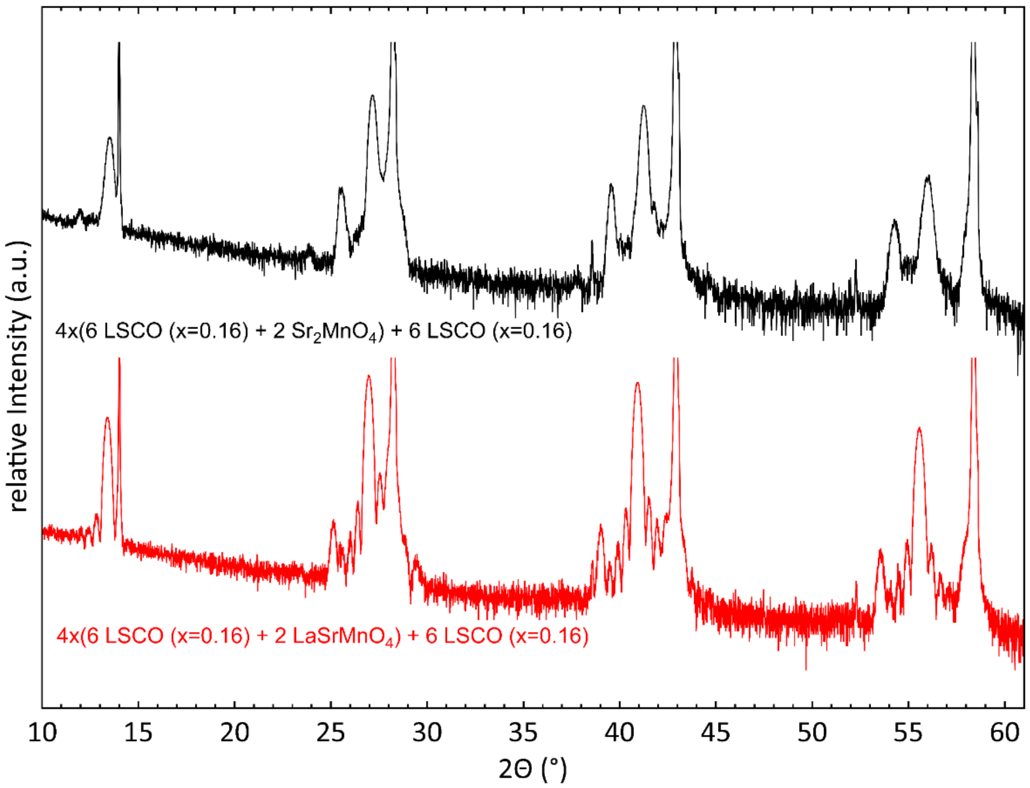


**SI Figure 1.** X-ray diffraction of both SLs. Top and bottom diffractogram corresponds to the SL with SMO and LSMO, respectively.

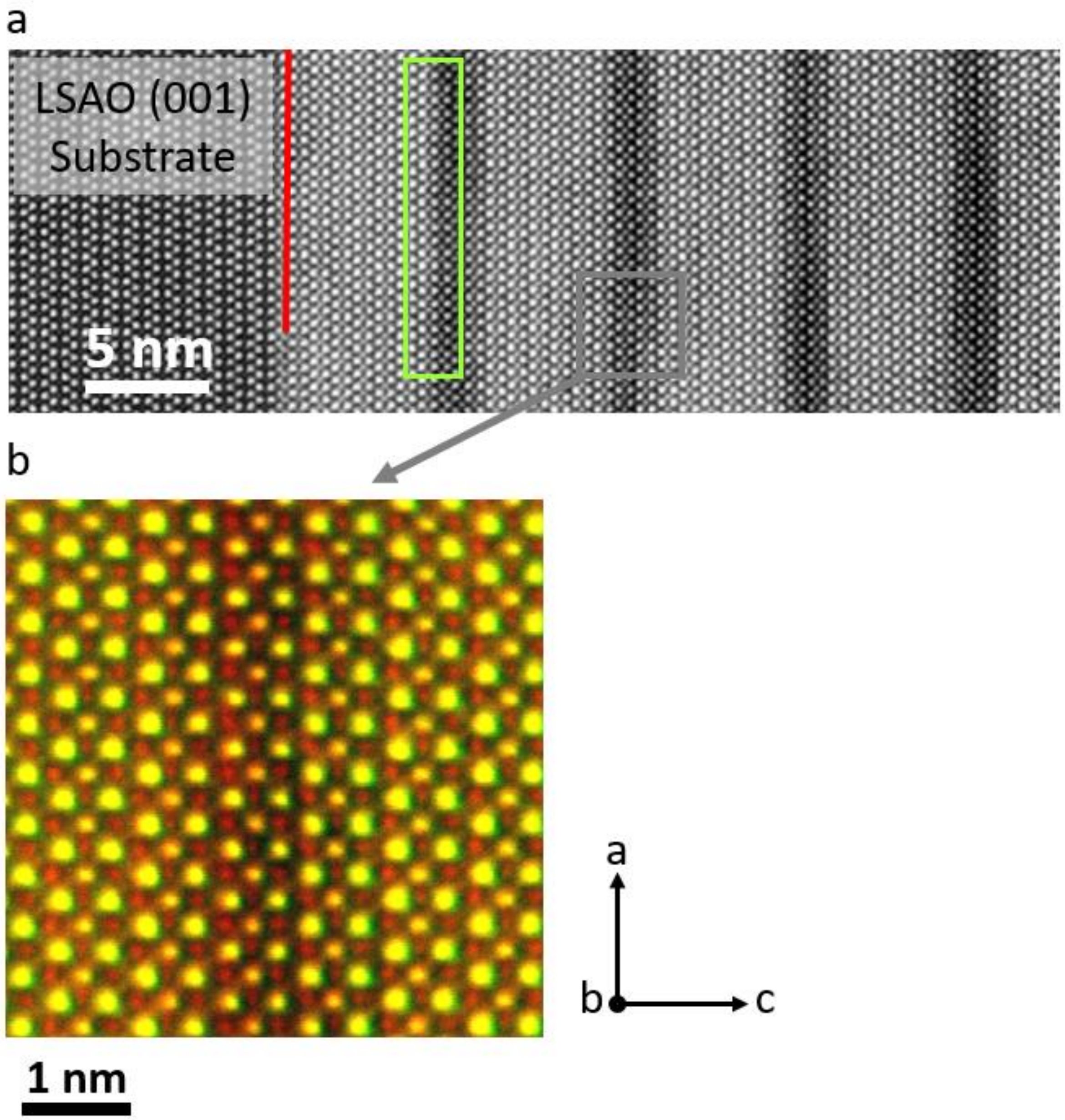


**SI Figure 2.** Overview of the superlattice with $Sr_2MnO_4$. (**a**) ADF overview image of the SL consisting of LSCO and SMO. Red bar points out the interface from the substrate to the first LSCO layer. The green box highlights the formation of a 113-type layer in the SMO area. (**b**) Overlay of high angle annular dark field and inverted annular bright field images.

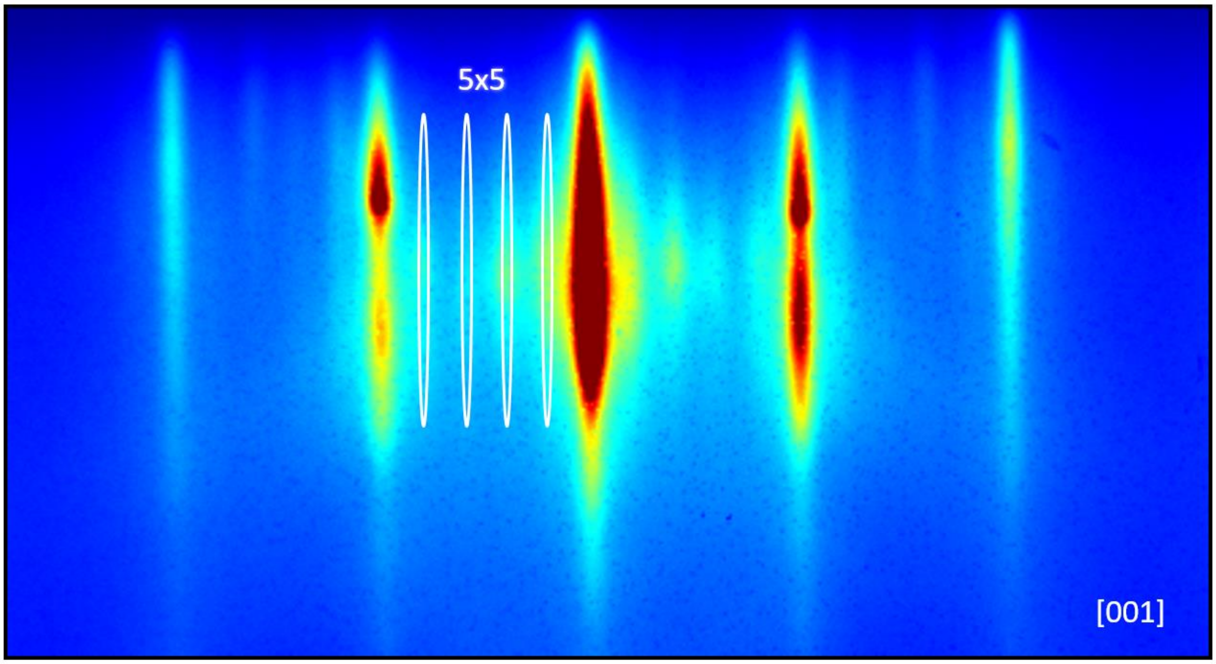


**SI Figure 3**. RHEED pattern of the first unit cell LSCO after the LSMO phase highlighting the 5x5 superstructure with four white ellipses.

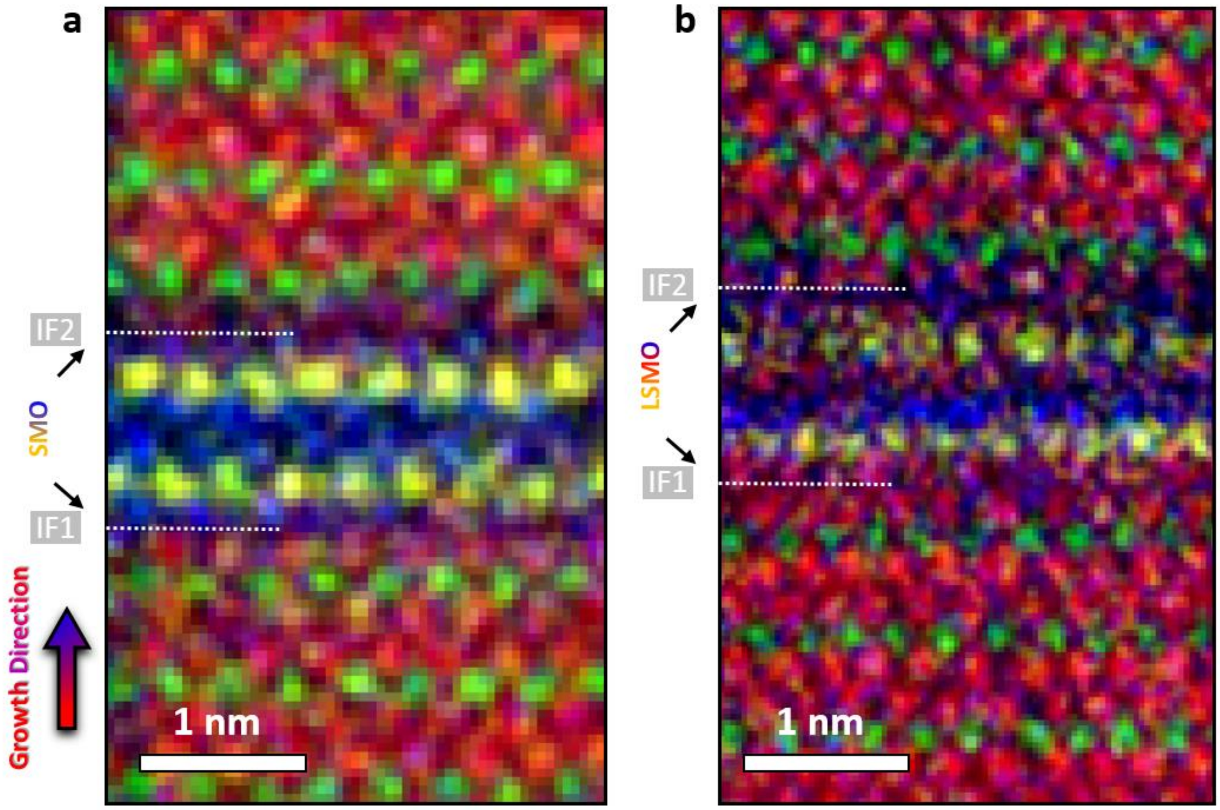


**SI Figure 4**. EELS elemental mapping obtained from raw data. (**a**) and (**b**) SLs with SMO and LSMO, respectively. Black arrows highlight the two interfaces (IF1 and IF2).

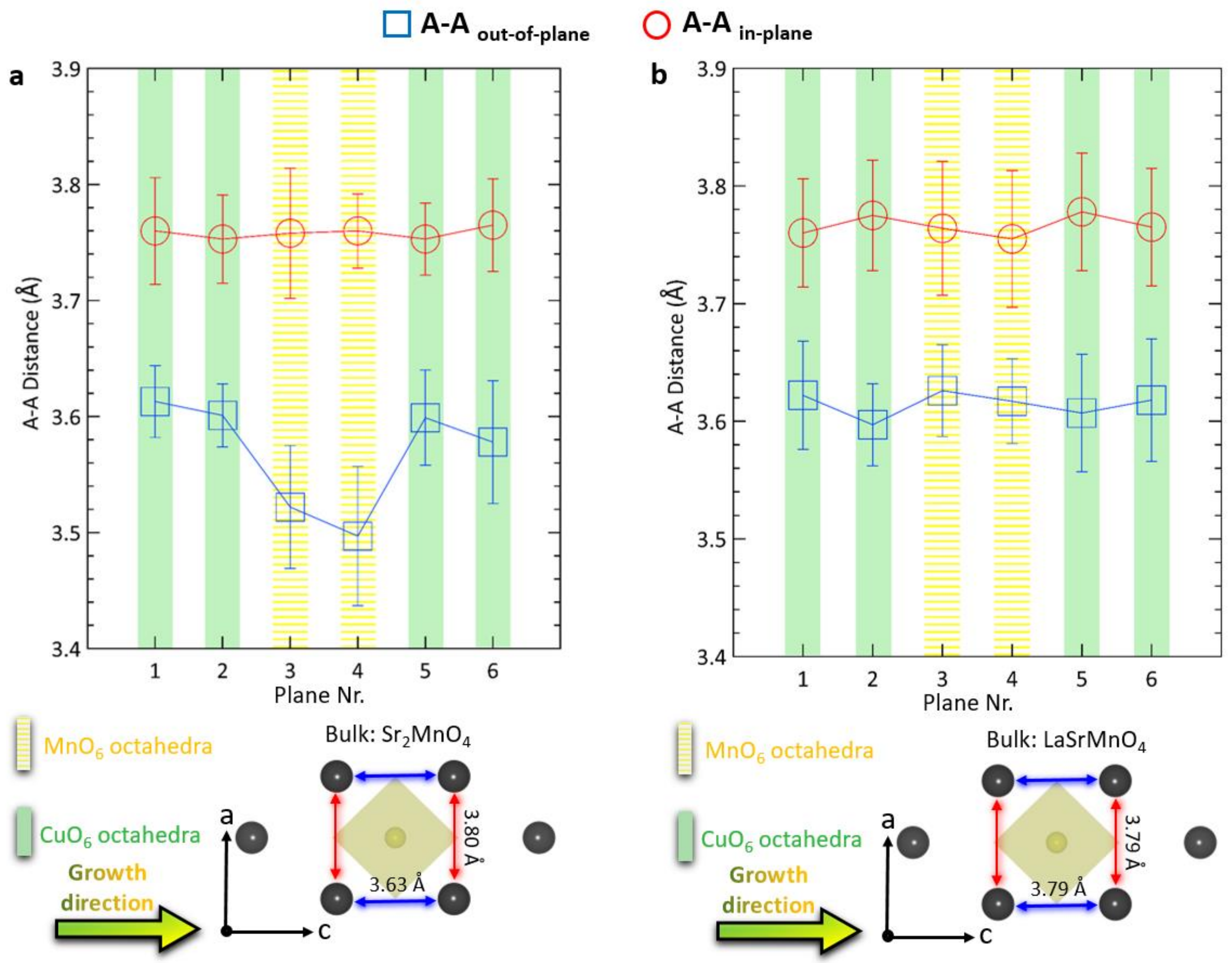


**SI Figure 5**. Out-of-plane (blue boxes) and in-plane (red circles) A-site cation distances obtained from HAADF images for the SL with SMO (**a**) and LSMO (**b**). The green arrow points out the growth direction and the red and blue arrows depict the out-of-plane and in-plane distances for SMO and LSMO bulk material. Error bars arise from two times the standard deviation of atomic-column positions within the same plane.
